# Measurement Accuracy of Freezing of Gait Scoring Based on Videos

**DOI:** 10.3389/fnhum.2022.828355

**Published:** 2022-05-19

**Authors:** Yuki Kondo, Katsuhiro Mizuno, Kyota Bando, Ippei Suzuki, Takuya Nakamura, Shusei Hashide, Hideki Kadone, Kenji Suzuki

**Affiliations:** ^1^Doctoral Programs in Intelligent and Mechanical Interaction Systems, University of Tsukuba, Tsukuba, Japan; ^2^Department of Physical Rehabilitation, National Center Hospital, National Center of Neurology and Psychiatry, Tokyo, Japan; ^3^Department of Rehabilitation Medicine, Keio University School of Medicine, Tokyo, Japan; ^4^Department of Rehabilitation Medicine, Tokai University School of Medicine, Kanagawa, Japan; ^5^Center for Innovative Medicine and Engineering, University of Tsukuba, Tsukuba, Japan; ^6^Center for Cybernics Research, University of Tsukuba, Tsukuba, Japan; ^7^Faculty of Engineering, Information and Systems, University of Tsukuba, Tsukuba, Japan

**Keywords:** Parkinson’s disease, freezing of gait, gait disorders, video-based detection, reliability

## Abstract

Freezing of gait (FOG) is a common symptom in the late stages of Parkinson’s disease and related disorders. Videos are the gold standard method to conduct FOG scoring; however, the measurement accuracy of FOG scoring based on videos has not been formally assessed, despite its use in previous studies. This study aimed to calculate the measurement accuracy of video-based FOG scoring. Three evaluators scored the FOG based on 157 video data points collected from 21 patients using an annotation tool. One evaluator measured the intra-rater reliability of the retest. The total duration of observed FOG, percentage of the time spent with FOG during the walking task (%FOG), and FOG phenotypes (shuffling, trembling, and complete akinesia) were evaluated. Intraclass correlation coefficients were used to determine the intra- and inter-rater reliabilities. The duration of FOG and %FOG showed good measurement accuracy for both intra-rater and inter-rater reliabilities. However, the FOG phenotypes showed poor measurement accuracy in inter-rater reliability. These results indicate that the temporal characteristics of FOG can be scored with a high degree of measurement accuracy, even with different evaluators; conversely, the FOG phenotypes need to be scored by several evaluators.

## Introduction

Freezing of gait (FOG) is commonly observed in Parkinson’s disease and related disorders. It is defined as “brief, episodic absence, or marked reduction of forward progression of the feet despite the intention to walk” (Nutt et al., 2011). In a previous systematic review and meta-analysis, the prevalence rate of FOG was reported to be 50.6% and gradually increased with disease progression (Zhang et al., 2021). In addition, FOG often leads to falls, fall-related injuries, and loss of independence (Bloem et al., 2004; Allen et al., 2013); hence, it may be an aggressive target for treatment.

The treatment of FOG is drug therapy (Schaafsma et al., 2003; Fietzek et al., 2013; Nonnekes et al., 2015); however, rehabilitative intervention and surgical treatment (deep brain stimulation) can also be used in combination to reduce symptoms (Schlenstedt et al., 2017; Cosentino et al., 2020). From a clinician’s and researcher’s point of view, assessing FOG is essential to initiate or change treatment and evaluate its effect.

Confirming any effective treatment would also require accurate measurement of FOG (Mancini et al., 2019; Cui and Lewis, 2021). The subjective methods of FOG assessment, such as history taking, the Unified Parkinson’s Disease Rating Scale, and special questionnaires [e.g., Freezing of Gait Questionnaire (Giladi et al., 2000) and New Freezing of Gait Questionnaire (Nieuwboer et al., 2009)], depend on the awareness of FOG among patients and caregivers. Therefore, clinicians and researchers cannot observe the occurrence of FOG. Furthermore, it has been reported that using the New Freezing of Gait Questionnaire findings, which are typified by subjective assessment, as a primary outcome in clinical trials is unsuitable because the two responses of the same respondent are inconsistent (Hulzinga et al., 2020). In addition, these subjective methods lack validation of the time of onset and duration of events.

Meanwhile, objective methods of FOG assessment, such as the use of videos and sensors, can help observe FOG, without the results being affected by recall bias. Therefore, it is beneficial to use objective methods to assess the effects of FOG treatment. Currently, FOG scoring based on video-recorded walking tasks is increasingly being recognized as the gold standard method for measuring the actual severity of FOG (Morris et al., 2012, 2013; Shine et al., 2012; Gilat, 2019). In addition, FOG scoring is consistent between intra- and inter-raters (Schaafsma et al., 2003; Morris et al., 2012). It is important to score FOG using this objective method, which currently requires the observation of FOG by trained clinicians per video. However, the calculation of the absolute measurement accuracy of FOG scoring using video has, to the best of our knowledge, not been reported by employing a method such as minimal/smallest detectable change (MDC/SDC). It is therefore essential to calculate the measurement accuracy of FOG scoring based on videos in order to ensure clinical interpretability before FOG scoring based on videos can be used in FOG research and clinical practice.

Therefore, the aim of this study was to calculate the measurement accuracy of video-based FOG scoring.

## Materials and Methods

### Evaluators

Three physical therapists specializing in rehabilitation for movement disorders at the National Center of Neurology and Psychiatry (NCNP) in Japan scored the FOG. Their years of practical experience as physical therapists were 7 (YK, evaluator A), 14 (KB, evaluator B), and 17 years (IS, evaluator C).

### Video Data Collection

This was a retrospective, single-center study. We collected video data recorded during clinical practice at the NCNP between April 2012 and March 2021. The recorded video data included the following: (1) clinical findings of Parkinson’s disease and related disorders; (2) conduct of the Timed Up and Go (TUG) test to provoke FOG (Shine et al., 2012); and (3) occurrence of FOG. A total of 157 video data points were collected from 21 patients, 13 of whom had Parkinson’s disease and eight had progressive supranuclear palsy. There were seven men and 14 women with a mean ± standard deviation age of 69.4 ± 8.8 years and disease duration of 11.4 ± 8.2 years. Five patients were in stage 3 and 16 patients were in stage 4 of the disease according to Hoehn and Yahr clinical staging. The study was approved by the institutional review board of the NCNP, Japan (approval number A2021-062) and conducted in accordance with the guidelines of the ethics committee of the NCNP and the Declaration of Helsinki.

### Freezing of Gait Scoring Based on Videos

We utilized a free template created by Gilat, which can be implemented in an open-source software to score FOG based on videos (Gilat, 2019). This software is called ELAN (version 6.0), which is a tool for creating complex annotations on video and audio recordings. The FOG scoring template available for use in ELAN contains predefined annotations that are frequently used to score FOG in research and clinical practice. This template, along with the user guide on how to measure the percentage of the time spent with FOG during the walking task (%FOG), can be downloaded for free.

The total duration of the TUG test, FOG, and FOG phenotypes was scored by the three evaluators based on videos. Each evaluator read and interpreted the definition of FOG by Nutt et al. (2011) and performed FOG scoring independently. Furthermore, the evaluators did not offer any input regarding the assessment of the video recordings. A total of 157 videos from 21 patients were presented to each rater in the same sequence. On completion, evaluator A distinguished the 157 videos in which each evaluator scored FOG based on the sub-types of FOG described by Fahn (Fahn, 1995): (i) hesitation to start; (ii) hesitation to turn; (iii) apparent hesitation in tight spaces; (iv) hesitation at destination; and (v) hesitation in open spaces; moreover, the percentage of each sub-type was calculated. Incidentally, the percentages of FOG in apparent hesitation in tight quarters were calculated by passing through a narrow passage task (37 videos), not from all videos. Only evaluator A scored the same items twice for the intra-rater evaluation. The interval between the two scorings was 2 weeks. The %FOG was calculated from the total duration of the FOG observed and the TUG test using the following formula (1).


(1) $$\%\mathrm{FOG}=\frac{\mathrm{Total}\mathrm{duration}\mathrm{of}\mathrm{FOG}\mathrm{observed}\mathrm{during}\mathrm{the}\mathrm{TUG}\mathrm{test}*100}{\mathrm{Total}\mathrm{duration}\mathrm{it}\mathrm{took}\mathrm{the}\mathrm{participant}\mathrm{to}\mathrm{perform}\mathrm{the}\text{TUG}\mathrm{test}}$$

The percentage of each FOG phenotype among the observed FOG was calculated.

The strength of FOG and %FOG is that they are an objective outcome of the ratio measurement level that directly reflects the severity of FOG at the time of testing as opposed to subjective scales such as freezing of gait questionnaires (Gilat, 2019). Therefore, we believe that it is appropriate to use the duration of FOG and %FOG to determine the effectiveness of treatment.

### Definition of Freezing of Gait

FOG was defined as “brief, episodic absence, or marked reduction of forward progression of the feet despite the intention to walk” (Nutt et al., 2011). The start time was defined as “the moment when the foot of the participant is suddenly no longer producing an effective step forward and displays FOG-related features (shuffling, trembling, and complete akinesia), despite the participant’s intention to continue walking.” Meanwhile, the end time was defined as “the moment of initial toe-off after FOG when the participant is again able to perform at least two effective alternating steps with both legs showing no FOG-related features” (Nutt et al., 2011; Gilat, 2019).

**FIGURE 1:**
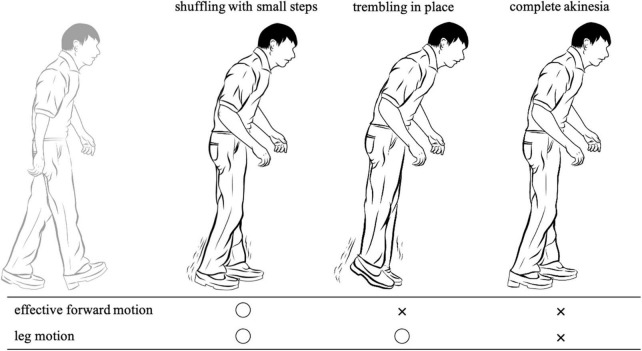
Three phenotypes of freezing of gait according to leg movements.

**FIGURE 2:**
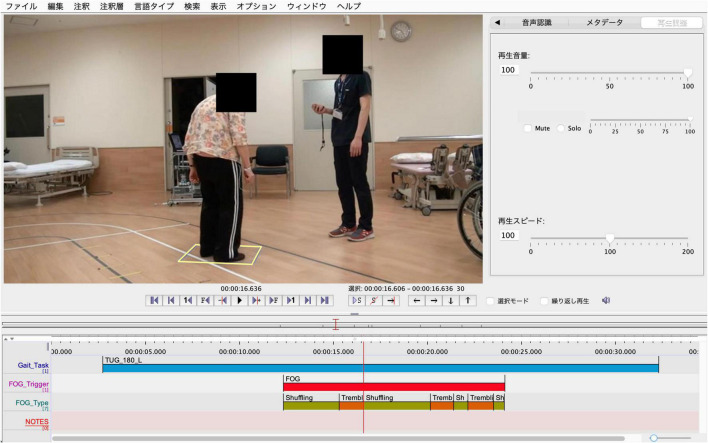
Sample utilizing ELAN. The evaluators watched the video and indicated the total duration of the TUG test (Gait_Tak), FOG (FOG_Trigger), and FOG phenotypes (FOG_Types), resulting in a TUG tag (blue rectangle), FOG tag (red rectangle), and FOG phenotype tag (shuffling = green rectangle; trembling = orange rectangle; complete akinesia = pink rectangle), respectively. These could be resized by dragging the ends horizontally in time by holding down the “option” key. TUG, timed up and go; FOG, freezing of gait.

Furthermore, the observed FOG was divided into the following three phenotypes according to leg movements (Figure 1): (i) FOG associated with very small shuffling steps and with minimal forward movement (shuffling), (ii) FOG with some leg trembling but no effective forward motion (trembling), and (iii) no observable motion of the legs (complete akinesia). The leg motion of each episode was scored and recorded according to the classification by Schaafsma et al. (2003). Figure 2 shows a sample that utilizes ELAN.

### Data Analyses

The SPSS software (version 27.0 for Mac) was used to evaluate intraclass correlation coefficients (ICC) after confirming a normal distribution using Shapiro–Wilk test. The intra-rater reliability for the first and second scores by evaluator A for the total duration of the FOG, %FOG, and FOG phenotypes was calculated using ICC_(1,1)_ with 95% confidence intervals (CIs). Similarly, the inter-rater reliability for the first assessment by evaluator A and the other two evaluators (B and C) for the total duration of the FOG, %FOG, and FOG phenotypes was calculated using ICC_(2,1)_ with 95% CIs. For the interpretation of ICC_(1,1)_ ranging from 0.00 to 1.00, values greater than 0.75 were considered to have good reliability (Koo and Li, 2016).

The standard error of measurement (SEM) (Chinn, 1991; Hopkins, 2000; Weir, 2005) (2), MDC/SDC (Portney and Watkins, 2009) (3), and technical error of measurement (TEM) (Goto and Mascie-Taylor, 2007) (4) were calculated using the following formula:


$$\text{SEM}=\frac{\text{SDd}}{\sqrt{2}}$$

(2) *SDd:thestandarddeviationofthedifferencescores

(3) $$\mathrm{MDC}/\mathrm{SDC}=1.96\times \sqrt{2}\times \mathrm{SEM}$$

(4) $$\text{TEM}=\sqrt{\frac{\sum_{i=1}^{N}{\left(x_{i1}-x_{i2}\right)}^{2}}{2N}}$$

$$*x_{\mathrm{in}}:\mathrm{score}\mathrm{of}\mathrm{the}\mathrm{nth}\mathrm{time}\mathrm{scored}\mathrm{by}\mathrm{evaluator}A$$

These parameters were used to represent measurement accuracy. The TEM was calculated only for the intra-rater reliability evaluation because scoring twice by the same evaluator is required.

## Results

### Video Data

The 157 videos consisted of 85 videos with patients performing a 180 degree turn, 35 videos with a 540 degree turn, and 37 videos with patients passing through a narrow passage. The percentages of FOG sub-types included in videos that were scored for FOG by evaluators A, B, and C were (i)17, 23, and 15% (of 157 total videos) in start hesitation; (ii) 91, 90, and 89% (of 157 total videos) in turn hesitation; (iii)100, 100, and 100% (of 37 videos when passing through a narrow passage) in apparent hesitation in tight quarters; (iv) 24, 28, and 22% (of 157 total videos) in destination-hesitation; and (v) 13, 12, and 11% (of 157 total videos) in open space hesitation, respectively.

### Intra-Rater Reliability

The descriptive statistics of the 157 videos of 21 patients for the intra-rater and inter-rater reliability evaluations are shown in Table 1. The ICC_(1,1)_ for the intra-rater reliability, SEM, MDC/SDC, and TEM for the total duration of the FOG, %FOG, and FOG phenotypes are presented in Table 2. The ICC_(1,1)_ for the total duration of FOG, %FOG, and FOG phenotypes was 1.00 (95% CI, 0.99–1.00), 0.99 (95% CI, 0.99–1.00), and 0.82–0.99, respectively.

**TABLE 1:** Freezing of gait (FOG) scoring by each evaluator (157 video data collected from 21 patients).

Evaluator	A_1st	A_2nd	B	C
FOG (s)	23.0 ± 30.7	23.3 ± 30.7	22.6 ± 30.5	20.5 ± 30.6
%FOG (%)	47.4 ± 22.7	48.3 ± 22.4	46.4 ± 22.4	39.5 ± 24.1
FOG phenotype				
Shuffling (%)	64.2 ± 31.8	56.9 ± 33.0	93.6 ± 14.4	67.1 ± 32.4
Trembling (%)	35.4 ± 31.3	42.7 ± 32.6	4.6 ± 10.5	31.0 ± 31.5
Akinesia (%)	0.4 ± 3.5	0.4 ± 3.6	1.8 ± 10.7	1.9 ± 11.0

*The data are presented as numbers or means ± standard deviations. FOG, freezing of gait.*

**TABLE 2:** Intra-rater reliability.

	ICC_(1,1)_	95% CI		SEM	MDC/SDC	TEM
		Lower	Upper			
FOG (s)	1.00	0.99	1.00	0.6	1.6	0.6
%FOG (%)	0.99	0.99	0.99	2.0	5.5	2.1
FOG phenotype						
Shuffling (%)	0.83	0.77	0.87	12.6	35.0	13.6
Trembling (%)	0.82	0.76	0.87	12.6	35.0	13.6
Akinesia (%)	0.99	0.98	0.99	0.4	1.1	0.4

*ICC, intraclass correlation coefficient; CI, confidence interval; SEM, standard error of the measurement; MDC/SDC, minimal/smallest detectable change; TEM, technical error of measurement; FOG, freezing of gait.*

The SEM for the duration of the FOG, %FOG, and FOG phenotypes was 0.6 s, 2.0%, and 0.4–12.6%, respectively. The MDC/SDC for the duration of the FOG, %FOG, and FOG phenotypes was 1.6 s, 5.5%, and 1.1–35.0%, respectively. In addition, the TEM for the duration of the FOG, %FOG, and FOG phenotypes was 0.6 s, 2.1%, and 0.4–13.6%, respectively.

### Inter-Rater Reliability

The ICC_(2,1)_ for the inter-rater reliability, SEM, and MDC/SDC for the total duration of the FOG, %FOG, and FOG phenotypes is presented in Table 3. The ICCs_(2,1)_ for the total duration of FOG, %FOG, and FOG phenotypes was 0.99 (95% CI, 0.99–0.99), 0.89 (95% CI, 0.79–0.93), and 0.31–0.44, respectively.

**TABLE 3:** Inter-rater reliability.

	ICC_(2,1)_	95% CI		SEM	MDC/SDC
		Lower	Upper		
FOG (s)	0.99	0.99	1.00	2.5	6.9
%FOG (%)	0.89	0.79	0.93	6.6	18.3
FOG phenotype					
Shuffling (%)	0.34	0.14	0.51	20.1	55.7
Trembling (%)	0.31	0.11	0.49	19.7	54.6
Akinesia (%)	0.44	0.35	0.54	6.8	18.8

*ICC, intraclass correlation coefficient; CI, confidence interval; SEM, standard error of the measurement; MDC/SDC, minimal/smallest detectable change; FOG, freezing of gait.*

The SEM for the duration of the FOG, %FOG, and FOG phenotypes was 2.5 s, 6.6%, and 6.8–20.1%, respectively. The MDC/SDC for the duration of FOG, %FOG, and FOG phenotypes was 6.9 s, 18.3%, and 18.8–55.7%, respectively.

## Discussion

The purpose of this study was to determine the measurement accuracy of FOG scoring based on videos. We found that the duration of FOG and %FOG showed good intra-rater and inter-rater reliabilities. However, the FOG phenotypes showed a poor inter-rater reliability, indicating scoring of the FOG phenotypes without consistency.

### Measurement Accuracy for the Temporal Characteristics of Freezing of Gait

Our results indicated that the total duration of FOG and %FOG in FOG scoring based on videos had good intra-rater (ICC = 1.00 and 0.99) and inter-rater (ICC = 0.99 and 0.89) reliabilities because an ICC greater than 0.75 was considered good (Koo and Li, 2016). The percent of freezing was demonstrated to have intra- (mean ICC = 0.71) and inter-rater (ICC = 0.73) agreement in a previous study (Morris et al., 2012). The study showed that ICC values were slightly lower than those in the present study. Possible causes were that this study was conducted in a single center; moreover, the evaluators performed scoring in the same sequence.

The SEM, MDC/SDC, and TEM for the total duration of FOG and %FOG were found to have a low intra-rater reliability. The SEM is an indication of the precision of a score (Weir, 2005), which is the minimum amount of change in a measure unlikely to be attributed to chance variation in measurement and is interpreted in clinical studies as the minimum amount of change required to designate the change as real and beyond the bounds of measurement error (Haley and Fragala-Pinkham, 2006). The TEM is the variability encountered between dimensions when the same specimens are measured in multiple sessions (Harris and Smith, 2009). The values represent the measurement accuracy, and low values indicate a high measurement accuracy. In this study, we found that the MDC/SDC for the total duration of FOG was 1.6 s, and the % intra-rater reliability was 5.5% FOG. However, the MDC/SDC for the total duration of FOG was slightly higher for the inter-rater reliability, which was 6.9 s, while the %FOG was 18.3%; this indicates that a change in the total duration of FOG and %FOG should surpass 6.9 s and 18.3% to be beyond the measurement error, respectively. The total duration of FOG and %FOG in FOG scoring based on videos showed a high measurement accuracy in terms of the ICC, regardless of the evaluator. Meanwhile, the MDC/SDC for the total duration of FOG was higher for the inter-rater than for the intra-rater reliability; the measurement accuracy was guaranteed by scoring by the same evaluator. For all of these reasons, we recommend that the same evaluator should measure the total duration of FOG and %FOG when these items are used to evaluate the treatment effect in FOG.

### Efficacy of Freezing of Gait Phenotype Scoring

The FOG phenotypes showed poor measurement accuracy in the inter-rater evaluation. The sample size was sufficient because the COSMIN guidelines recommend that performing the analysis in 100 samples is very good (Prinsen et al., 2018). We assumed two reasons for the poor measurement accuracy. First, the definition of the FOG phenotypes may have been ambiguous. We utilized the definition by Schaafsma et al. and divided the three phenotypes of FOG according to leg movements (Schaafsma et al., 2003). It was difficult to determine whether this could be scored as shuffling or trembling using this definition. For example, if during a FOG episode, one leg was moving forward but the other leg still showed FOG-related features, selection was difficult for the evaluators. Second, a two-dimensional video provides less information than a three-dimensional gait analysis. It has been reported that errors occur when the step length is estimated from videos; in particular, the step length is estimated most inaccurately when the individual is not in the center of the field of view of the camera (Stenum et al., 2021). FOG phenotypes are determined by leg movements and whether the patient is moving forward (Schaafsma et al., 2003). Assessments using videos obscured the estimation of whether the feet were moving and whether the individual was moving forward, for example, wearing loose clothing. In addition, if a caregiver or chair overlaps with a patient, it is more difficult to score the FOG phenotypes. Because of the high ICCs for the intra-rater reliability, we believe that the ambiguous definition mentioned first was more plausible than the second reason. The recognition of FOG is well established within individual; however, recognition of FOG is divided among experts. Therefore, it might be even more difficult for patients to recognize FOG.

The results of the FOG phenotypes for the evaluators A and C were similar, whereas those for evaluator B were dissimilar. There were large differences among evaluators in identifying “shuffling” and “trembling.” Evaluator A and C recognized one leg moving forward with the other leg not moving, which is similar to a pivot turn, as “trembling,” while evaluator B recognized it as “shuffling.” Therefore, we believe that adding a supplementary explanation that emphasizes an effective step is reasonable for defining FOG phenotypes. For example, if one leg is moving forward but the other leg is not, it is not an effective step and should be determined as “trembling.” An important first step to precisely comprehend the definition of FOG phenotypes is discussion between researchers.

However, distinguishing the FOG phenotypes might be currently of low importance in clinical practice. Currently, there may be no clinical advantage in dividing the FOG phenotypes because changes in the treatment depending on these variables have not been reported, although it is known that FOG can be divided into three phenotypes according to leg movements (Schaafsma et al., 2003; Nutt et al., 2011). The study findings indicate that the measurement accuracy of FOG phenotype scoring is important and needs to be improved in case further studies reveal the clinical advantage of dividing FOG phenotypes in the future. Therefore, it may be too early to use the FOG phenotypes as a primary outcome in research and clinical practice.

### Limitations

This study was conducted in a single center. We hypothesized that there would be more variation in the results because different countries have different languages and may have different interpretations of the definition of FOG. Future studies are needed to examine whether the results are consistent, even among experts and centers.

We plan to develop video-based methods for automatic FOG detection. Several recent studies have been conducted to develop automatic methods for detecting and assessing FOG based on videos (Hu et al., 2019, 2020; Cao et al., 2021). These systems can be divided into freezers and non-freezers, although they still cannot quantify the duration of FOG and %FOG. Based on the results of this study, we plan to develop a highly accurate system for automatic FOG detection based on videos in future research by (i) utilizing only the total duration of FOG and FOG% (even when experts made the decision, they had different opinions) and (ii) creating labeled training data after several evaluators discuss and correct the video data with high variability.

## Conclusion

In conclusion, the duration of FOG and %FOG demonstrated good measurement accuracy in both intra-rater and inter-rater evaluations. However, the FOG phenotypes showed poor measurement accuracy in inter-rater evaluation. Caution is warranted when scoring the FOG phenotypes based on videos as a primary outcome in research and clinical practice. Several evaluators are necessary to score FOG phenotypes. Evaluation of the measurement accuracy of FOG scoring based on videos will contribute to the advancement of research and clinical practice for Parkinson’s disease and related disorders.

## Data Availability Statement

The raw data supporting the conclusions of this article will be made available by the authors, without undue reservation.

## Ethics Statement

The studies involving human participants were reviewed and approved by the Institutional Review Board of the National Center of Neurology and Psychiatry. The patients/participants provided their written informed consent to participate in this study.

## Author Contributions

YK: conceptualization, methodology, data correction, formal analysis, and writing – original draft. KM, TN, and SH: recruiting, and writing – review and editing. KB and IS: data correction and formal analysis. HK: writing – review and editing. KS: conceptualization, methodology, and writing – review and editing. All authors contributed to the article and approved the submitted version.

## Conflict of Interest

The authors declare that the research was conducted in the absence of any commercial or financial relationships that could be construed as a potential conflict of interest.

## Publisher’s Note

All claims expressed in this article are solely those of the authors and do not necessarily represent those of their affiliated organizations, or those of the publisher, the editors and the reviewers. Any product that may be evaluated in this article, or claim that may be made by its manufacturer, is not guaranteed or endorsed by the publisher.
